# Elesclomol elevates cellular and mitochondrial iron levels by delivering copper to the iron import machinery

**DOI:** 10.1016/j.jbc.2022.102139

**Published:** 2022-06-14

**Authors:** Natalie M. Garza, Mohammad Zulkifli, Vishal M. Gohil

**Affiliations:** Department of Biochemistry and Biophysics, MS 3474, Texas A&M University, College Station, Texas, USA

**Keywords:** Copper, iron, mitochondria, Golgi, Fet3, Ccc2, ES, elesclomol, ES-Cu, elesclomol-copper, GRX1, glutaredoxin-1, ICP-MS, inductively coupled plasma-mass spectrometry

## Abstract

Copper (Cu) and iron (Fe) are redox-active metals that serve as cofactors for many essential cellular enzymes. Disruption in the intracellular homeostasis of these metals results in debilitating and frequently fatal human disorders, such as Menkes disease and Friedreich’s ataxia. Recently, we reported that an investigational anticancer drug, elesclomol (ES), can deliver Cu to critical mitochondrial cuproenzymes and has the potential to be repurposed for the treatment of Cu deficiency disorders. Here, we sought to determine the specificity of ES and the ES-Cu complex in delivering Cu to cuproenzymes in different intracellular compartments. Using a combination of yeast genetics, subcellular fractionation, and inductively coupled plasma-mass spectrometry–based metal measurements, we showed that ES and ES-Cu treatment results in an increase in cellular and mitochondrial Fe content, along with the expected increase in Cu. Using yeast mutants of Cu and Fe transporters, we demonstrate that ES-based elevation in cellular Fe levels is independent of the major cellular Cu importer but is dependent on the Fe importer Ftr1 and its partner Fet3, a multicopper oxidase. As Fet3 is metalated in the Golgi lumen, we sought to uncover the mechanism by which Fet3 receives Cu from ES. Using yeast knockouts of genes involved in Cu delivery to Fet3, we determined that ES can bypass Atx1, a metallochaperone involved in Cu delivery to the Golgi membrane Cu pump, Ccc2, but not Ccc2 itself. Taken together, our study provides a mechanism by which ES distributes Cu in cells and impacts cellular and mitochondrial Fe homeostasis.

Copper (Cu) is an essential micronutrient that is utilized as a cofactor for a diverse array of enzymes involved in different physiological processes (1). For example, Cu plays an essential and evolutionarily conserved role in mitochondrial energy generation, free radical detoxication, and iron (Fe) transport (1). In addition to these fundamental cellular processes, the redox property of Cu is harnessed into more specialized functions such as melanin production, collagen formation, and catecholamine biosynthesis in evolutionarily advanced organisms including humans (1). However, Cu is also highly reactive and can generate deleterious reactive oxygen species (2). Therefore, a dedicated set of Cu transporters and chaperones are present in cells to ensure optimal distribution and homeostasis of Cu while avoiding its toxicity (3, 4).

Genetic mutations that impair Cu absorption or its transport to Cu-containing enzymes (cuproenzymes) often manifest in fatal disorders (5, 6, 7, 8, 9, 10, 11). For example, mutations in Cu chaperones that are required for Cu delivery to a mitochondrial cuproenzyme, cytochrome *c* oxidase, result in mitochondrial disorders that typically present as fatal neurological and cardiac defects in infants (5, 9, 11, 12). Similarly, loss-of-function mutations in ATP7A, a protein required for dietary Cu absorption and its transport through the blood–brain barrier, result in a fatal neurological disorder called Menkes disease (10, 13, 14, 15). Currently, no FDA-approved treatments are available for these lethal disorders (16).

Recently, we showed that elesclomol (ES), a Cu-binding investigational oncology drug, can traverse through cellular membranes and make Cu bioavailable to mitochondrial cytochrome *c* oxidase and rescue Cu-deficient phenotypes in yeast, zebrafish, and murine models (17, 18). These exciting findings have raised the possibility of repurposing this cancer drug for the treatment of Cu deficiency disorders (19). ES is a bis (thiohydrazide) amide compound that was originally developed as an anticancer drug. Mechanistically, ES binds Cu(II) in a 1:1 ratio in the extracellular environment (20), forming a membrane permeable complex (ES-Cu), which upon entering the mitochondria, releases Cu by an unknown mechanism (21). In this manner, ES selectively kills cancer cells by transporting excess Cu to mitochondria and thereby inducing the mitochondrial apoptosis pathway (21, 22, 23).

Although previous work has established the Cu-specific role of ES (21) and its ability to deliver Cu to mitochondrial cuproenzyme (17, 18), we currently do not know how ES treatment impacts the levels of other metals in cells and whether it is able to deliver Cu to cuproenzymes in other (non-mitochondrial) subcellular compartments. A previous genome-wide study has shown that perturbation in the levels of one metal tend to affect the levels of other metals in cells (24). Moreover, ligands that bind Cu(II) can often bind Fe. Indeed, a mass spectrometry study reported that ES can also bind Fe(II) *in vitro* (25). These reports motivated our present study, where we show that in addition to the expected elevation in cellular and mitochondrial Cu content, treatment with ES also increases cellular Fe content indirectly by stimulating Cu-dependent Fe import machinery.

## Results

### Preformed ES-Cu complex is more efficient than ES in transporting Cu

**Figure 1:**
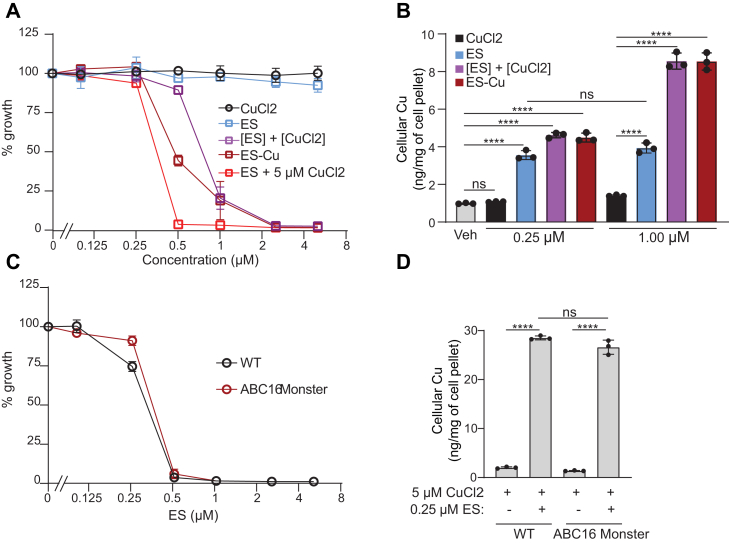
**Determination of the maximal tolerable dose of ES and ES-Cu complex.***A*, BY4741 WT yeast cells were cultured in YPD medium at 30 °C in the presence of increasing concentrations (0.1–5 μM) of the indicated compound. The cell density was measured spectrophotometrically after 12 h of growth at 600 nm. Percent of growth was calculated by comparing the optical density of each culture to that of WT treated with vehicle. *B*, Cu levels in BY4741 WT yeast cells treated with the indicated compound were measured by ICP-MS. *C*, ABC_16_ monster yeast cells and their isogenic WT control were cultured at 30 °C in YPD + 5 μM CuCl_2_ with increasing concentrations (0.1–5 μM) of ES. The cell density was measured spectrophotometrically after 12 h of growth at 600 nm. *D*, Cu levels in WT and ABC_16_ yeast cells, grown at 30 °C in YPD + 5 μM CuCl_2_ supplemented with either vehicle or 0.25 μM ES, were measured by ICP-MS. Data are expressed as mean ± SD; (n = 3), ∗∗∗∗*p* < 0.0001, ns = not significant. ES, elesclomol; ES-Cu, elesclomol-copper complex; ICP-MS, inductively coupled plasma-mass spectrometry.

Previous studies have established that cytotoxic effects of ES are dependent on the availability of Cu in the growth media, which implies that ES transports external Cu into the cells (21, 26). As both ES and ES-Cu are promising molecules for treating inborn errors of Cu metabolism (17, 18), we first aimed to compare and quantify the toxicity and efficiency of these compounds as Cu couriers. We observed that the growth of WT yeast grown in glucose-containing media was minimally affected by the presence of either CuCl_2_ or ES at the highest concentration (5 μM) tested (Fig. 1*A*). However, when the growth media was supplemented with preformed ES-Cu complex or ES cosupplemented with CuCl_2_, we observed growth inhibition at concentrations above 0.25 μM, with inhibitory concentration (IC_50_) values of ES-Cu being 0.57 μM and ES cosupplemented with equimolar CuCl_2_ being 0.82 μM (Fig. 1*A*). Notably, when increasing concentrations of ES were supplemented in the presence of excess (5 μM) CuCl_2_ in the media, we observed the most severe growth inhibition with an IC_50_ of 0.32 μM (Fig. 1*A*). These findings are consistent with previous studies (21, 26) and with the idea that ES shuttles in and out of the cells, continuously bringing extracellular Cu into the cells.

Next, we wanted to determine the intracellular Cu concentration that inhibits yeast growth. Supplementation of 0.25 μM or 1 μM CuCl_2_ did not significantly increase intracellular Cu abundance (Fig. 1*B*). This could be due to reduced expression of Ctr1, the major Cu importer, in Cu replete conditions (27). In contrast to CuCl_2_, supplementation of 0.25 μM of ES, ES + CuCl_2_ (each added separately in the media), or preformed ES-Cu complex increased intracellular Cu content by ∼4 fold (Fig. 1*B*). Furthermore, we found that treatment with ES-Cu or ES + CuCl_2_ increased cellular Cu content in a dose-dependent manner, but ES alone did not (Fig. 1*B*), which is likely due to limited bioavailable Cu in the media. These data suggest that yeast growth is not impaired with a ∼4-fold increase in intracellular Cu levels, but 8-fold increase in Cu levels starts inhibiting cellular growth (Fig. 1, *A* and *B*).

It has been previously suggested that upon unloading Cu in mitochondria, ES is exported out of the cells by a p-glycoprotein drug pump, whereby it can bring in more Cu from extracellular compartments (21). To test this idea and to identify the drug pump responsible, we used an ABC_16_ monster yeast strain, which lacks 16 known ABC transporters associated with drug resistance (28). We expected that deletion of the putative ES-specific drug pump would decrease the sensitivity of the mutant to ES because it would “trap” ES inside the cells, preventing it from shuttling more extracellular Cu into the cells. Treatment of the WT and ABC_16_ monster yeast strain with increasing concentration of ES in the presence of excess (5 μM) CuCl_2_ showed only a marginal change in IC_50_ values between the WT and the mutant (0.26 μM WT *versus* 0.30 μM ABC_16_ mutant) (Fig. 1*C*). Consistent with this, we find that the amount of Cu accumulation in the WT and ABC_16_ mutant upon ES treatment was very similar (Fig. 1*D*). These results suggest that export of ES is not dependent on any of the 16 ABC transporters; however, we cannot rule out ES efflux through other transporters or simple diffusion through the plasma membrane.

### Effect of ES-Cu treatment on cellular metallome

**Figure 2:**
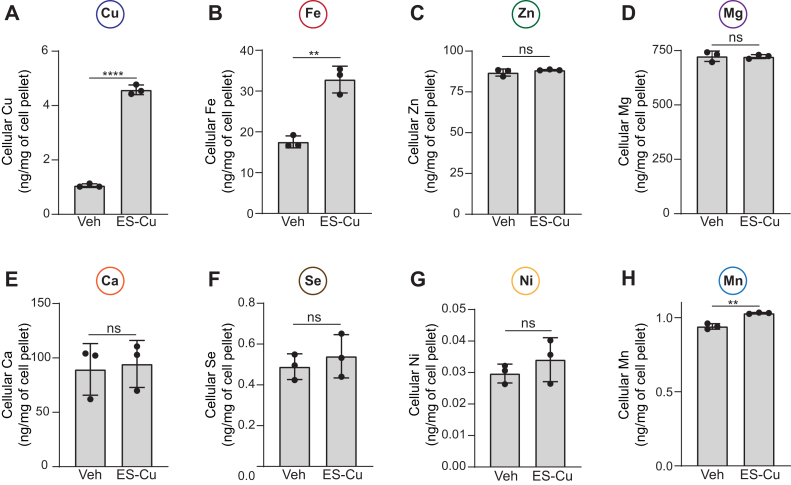
**ES-Cu treatment elevates cellular Fe levels.***A–H*, BY4741 WT cells were grown in YPD ± 0.25 μM ES-Cu for 10 h before measuring the cellular content of the indicated metals by ICP-MS. Data are expressed as mean ± SD; (n = 3), ∗∗*p* < 0.01, ∗∗∗∗*p* < 0.0001, ns = not significant. ES-Cu, elesclomol-copper complex; ICP-MS, inductively coupled plasma-mass spectrometry.

Since perturbation in the homeostasis of one metal often results in widespread changes in the cellular metallome (24), we sought to determine if ES-Cu supplementation altered the cellular abundance of other metals. For accurate measurements of intracellular metal content, samples were serially diluted, and tailored calibration curves were generated by inductively coupled plasma-mass spectrometry (ICP-MS) (Fig. S1). By using this method, we were able to detect cellular metal levels spanning over four orders of magnitude (Fig. S1). ES-Cu treatment led to an expected increase in intracellular Cu levels (Fig. 2*A*). In addition to Cu, we found a striking increase in intracellular Fe levels as well (Fig. 2*B*). The intracellular abundance of Zn, Mg, Ca, Se, and Ni were unaltered by treatment with ES-Cu (Fig. 2, *C*–*G*), although we did observe a small but significant increase in Mn levels (Fig. 2*H*). Notably, the increase in Cu and Fe levels upon ES-Cu treatment is independent of yeast strain background because we observed a similar increase in the levels of both these metals in a different yeast strain (W303) (Fig. S2). Together, these data suggest that ES and ES-Cu elevate intracellular levels of both Cu and Fe.

### ES and ES-Cu treatment elevates mitochondrial Fe

**Figure 3:**
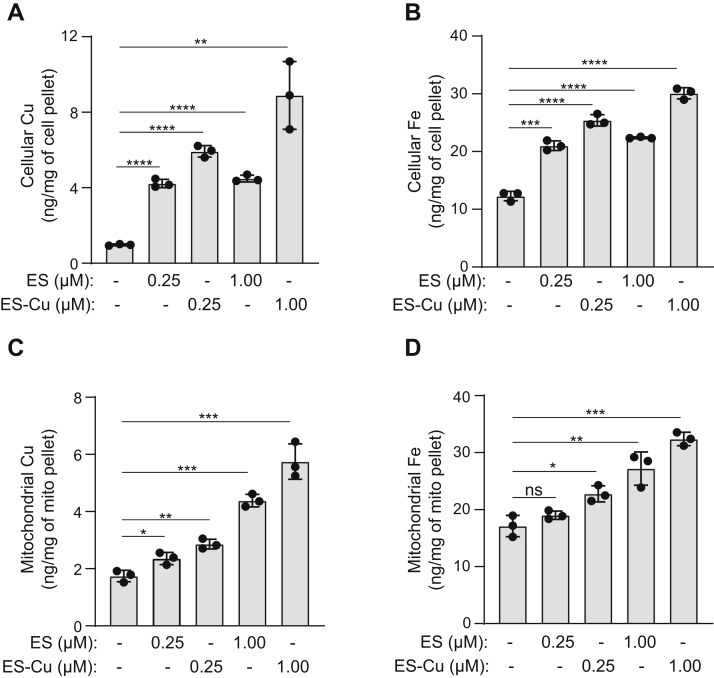
**ES and ES-Cu treatment elevates mitochondrial Fe levels.** BY4741 WT yeast cells were cultured in YPGE medium at 30 °C in the presence of 0.25 or 1 μM of either ES or ES-Cu. Cellular Cu (*A*) and Fe (*B*) levels and mitochondrial Cu (*C*) and Fe (*D*) levels were measured by ICP-MS. Data are expressed as mean ± SD; (n = 3), ∗*p* < 0.05, ∗∗*p* < 0.01, ∗∗∗*p* < 0.001, ∗∗∗∗*p* < 0.0001, ns = not significant. ES, elesclomol; ES-Cu, elesclomol-copper complex; ICP-MS, inductively coupled plasma-mass spectrometry.

Since mitochondria contain many Fe-dependent enzymes, we wanted to determine whether ES and ES-Cu treatment could increase Fe levels in mitochondria. We performed these experiments using nonfermentable growth media (YPGE), which is known to derepress mitochondrial biogenesis genes (29). As in YPD, cells cultured in YPGE were more sensitive to ES-Cu than ES alone, with IC_50_ of ES-Cu–mediated growth inhibition being 0.74 μM (Fig. S3). We were able to ascribe increased toxicity of ES-Cu to elevated levels of intracellular Cu (Fig. 3*A*). Both ES and ES-Cu treatment increased intracellular Cu and Fe levels albeit to a different degree (Fig. 3, *A* and *B*). Similar to an increase in cellular Cu and Fe, ES and ES-Cu also led to an increase in their levels in mitochondria (Fig. 3, *C* and *D*). This result shows that Fe imported in an ES- and ES-Cu–dependent manner is being transported to the mitochondria (Fig. 3*D*).

### ES-Cu–mediated increase in Fe levels is dependent on the high affinity Fe import machinery

**Figure 4:**
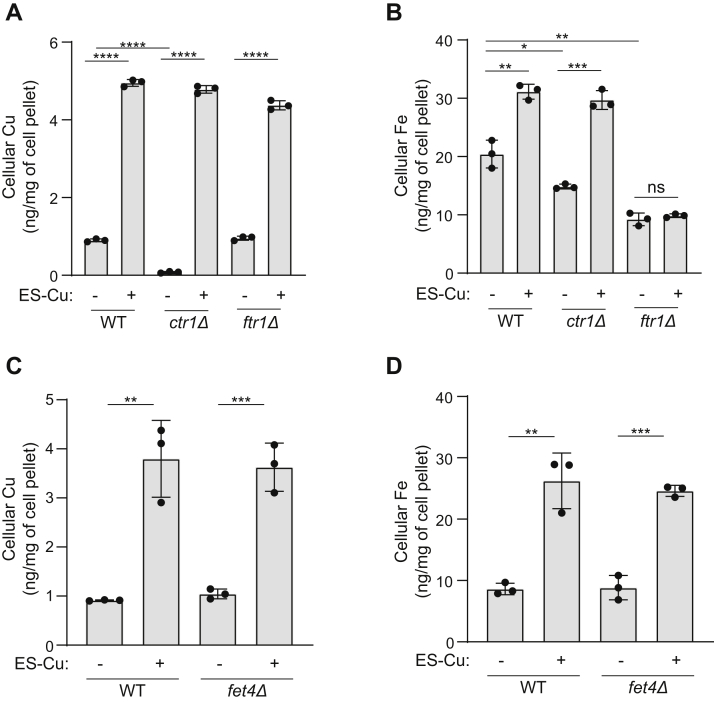
**ES-Cu–mediated increase in intracellular Fe content is dependent on the high affinity Fe import machinery.** BY4741 WT, *ctr1Δ*, and *ftr1Δ* cells were grown in YPD ± 0.25 μM ES-Cu for 10 h before measuring Cu (*A*) and Fe (*B*) by ICP-MS. BY4741 WT and *fet4Δ* cells were grown in YPD ± 0.25 μM ES-Cu for 10 h before measuring Cu (*C*) and Fe (*D*) levels by ICP-MS. Data are expressed as mean ± SD; (n = 3), ∗*p* < 0.05, ∗∗*p* < 0.01, ∗∗∗*p* < 0.001, ∗∗∗∗*p* < 0.0001, ns = not significant. ES-Cu, elesclomol-copper complex; ICP-MS, inductively coupled plasma-mass spectrometry.

Next, we wanted to determine the mechanism of ES-Cu–mediated elevation in cellular Fe levels. We considered the following two possibilities: first, ES is able to exchange Cu with extracellular Fe and transport it into cells in a manner similar to Cu; second, ES-Cu elevates cellular Fe indirectly by stimulating Fe import machinery. To distinguish between these possibilities, we measured the Fe and Cu contents of ES-Cu–treated *ctr1Δ* and *ftr1Δ* yeast mutants that are devoid of major Cu and Fe importers, respectively (27, 30). As expected, *ctr1Δ* cells exhibited reduced Cu levels and treatment with ES-Cu elevated cellular Cu levels in this mutant to the same extent as WT (Fig. 4*A*). Similar levels of Cu accumulation were observed in ES-Cu–treated *ftr1Δ* cells (Fig. 4*A*). These data demonstrate that increase in cellular Cu by ES is independent of Cu or Fe import machinery. In contrast, we found that ES-Cu treatment increased Fe levels only in WT and *ctr1Δ* cells but not in *ftr1Δ* cells (Fig. 4*B*). This data argues against direct transport of extracellular Fe by ES and indicates that ES-Cu–mediated increase in cellular Fe levels is dependent on Ftr1, the high affinity Fe importer. Notably, the loss of the low affinity Fe importer, Fet4 (31, 32) did not impact ES-Cu–mediated increase in Cu and Fe levels (Fig. 4, *C* and *D*). Together, these data show that Ftr1 is essential for ES-Cu–mediated increase in intracellular Fe.

### Fet3 and Ccc2 are essential for ES-Cu–mediated increase in cellular Fe levels

**Figure 5:**
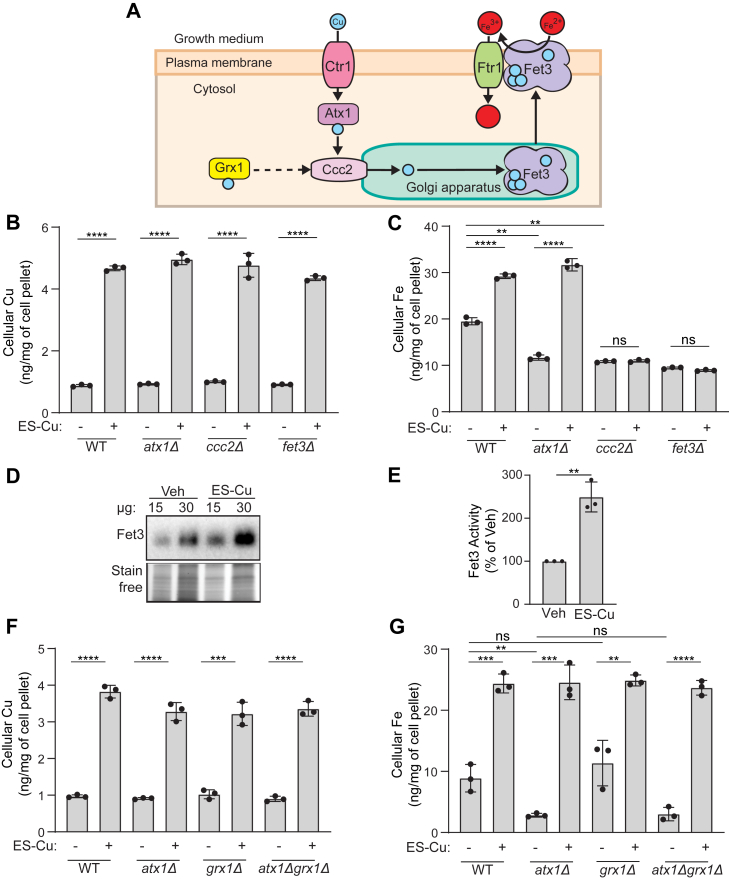
**ES-trafficked Cu is bioavailable to Ccc2 for its insertion into Fet3.***A*, a schematic of Cu transport to the Fe import machinery protein Fet3. Cu is imported *via* the high affinity Cu transporter Ctr1. Once inside the cell, Cu is bound by the Cu-metallochaperone Atx1, which transports Cu to Ccc2, a P-type ATPase present on the Golgi membrane that pumps Cu into the Golgi lumen where Fet3 metalation takes place. Metalated Fet3 is subsequently transported to the plasma membrane where it works in conjunction with Ftr1 to import extracellular Fe into cells. GRX1 has been suggested to transport Cu to Ccc2 homolog ATP7B, indicated by the *dashed line*. *B* and *C*, BY4741 WT, *atx1Δ*, *ccc2Δ*, and *fet3Δ* were grown in YPD ± 0.25 μM ES-Cu for 10 h before measuring Cu (*B*) and Fe (*C*) levels by ICP-MS. *D*, in-gel Fet3 oxidase activity assay performed on cellular extracts from vehicle or 0.25 μM ES-Cu–treated BY4741 WT cells. Stain-free imaging of the same gel was used as a loading control. *E*, quantification of Fet3 activity for 15 μg protein samples from panel (*D*). *F* and *G*, BY4741 WT, *atx1Δ*, *grx1Δ*, and *atx1Δgrx1Δ* cells were grown in YPD ± 0.25 μM ES-Cu for 10 h before measuring Cu (*F*) and Fe (*G*) levels by ICP-MS. Data are expressed as mean ± SD; (n = 3), ∗∗*p* < 0.01, ∗∗∗*p* < 0.001, ∗∗∗∗*p* < 0.0001, ns = not significant. ES, elesclomol; ES-Cu, elesclomol-copper complex; GRX1, glutaredoxin-1; ICP-MS, inductively coupled plasma-mass spectrometry.

Fet3 is a plasma membrane–localized multicopper oxidase that oxidizes Fe^2+^ to Fe^3+^ for its subsequent cellular uptake by Ftr1 (30, 33). Insertion of Cu into Fet3 occurs in the Golgi lumen and is essential for its activity (34) (Fig. 5*A*). Cytosolic Cu is pumped into the Golgi lumen by Ccc2, a P-type ATPase, which itself receives Cu from Atx1, a cytosolic Cu metallochaperone (Fig. 5*A*) (35, 36). To test if ES-Cu–mediated Fe import is dependent on these proteins (Atx1, Ccc2, and Fet3), we measured the Fe levels in cells lacking these proteins. As expected, treatment with ES-Cu led to a pronounced increase in intracellular Cu levels for each of these mutants (Fig. 5*B*). However, the increase in Fe levels was disrupted in *fet3Δ* and *ccc2Δ* mutants (Fig. 5*C*). In contrast, we found that Atx1 is dispensable for ES-Cu–mediated elevation in cellular Fe levels (Fig. 5*C*). We hypothesized that ES-Cu stimulated Fe import by making Cu available to Fet3 and thereby increasing its activity. Consistent with this hypothesis, we observed over two-fold increase in Fet3 enzymatic activity in cells treated with ES-Cu (Fig. 5, *D* and *E*). These data demonstrate that ES can bypass Atx1 but not Ccc2 in delivering Cu to Fet3 and suggest a possibility that another protein may compensate for the lack of Atx1.

Recently, an *in vitro* study showed that human glutaredoxin-1 (GRX1) can deliver Cu to one of the human homologs of Ccc2, ATP7B (37). This transfer of Cu was independent of ATOX1, the human homolog of Atx1. These findings suggested that GRX1 could substitute for ATOX1 function in Cu delivery to ATP7B. We, therefore, asked if the yeast homolog of human GRX1, Grx1, could substitute for Atx1 in delivering Cu to Ccc2 *in vivo* (Fig. 5*A*). To test this hypothesis, we constructed *grx1Δ* and *atx1Δgrx1Δ* yeast mutants. ICP-MS–based cellular metal measurements showed that the Cu levels of all mutants were comparable to the WT under basal or ES-Cu–supplemented conditions (Fig. 5*F*). However, Fe levels of these mutants varied under basal conditions, with *atx1Δ* and *atx1Δgrx1Δ* exhibiting reduced levels of intracellular Fe, whereas the Fe content of *grx1Δ* cells was comparable to that of WT (Fig. 5*G*). These results suggest that *in vivo*, Atx1 plays a critical role in supplying Cu to Golgi but Grx1 does not. Indeed, ES-Cu treatment led to an increase in Fe levels in both *grx1Δ* and *atx1Δgrx1Δ* cells (Fig. 5*G*). Together, these data demonstrate that ES-Cu can bypass both Atx1 and Grx1 in delivering Cu to Ccc2 *in vivo*.

## Discussion

**Figure 6:**
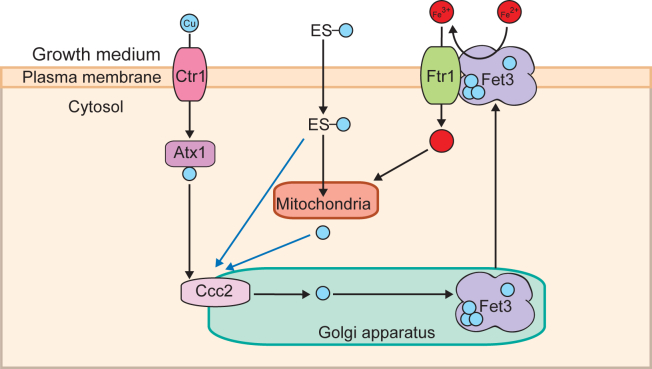
**A model depicting ES-Cu–mediated increase in mitochondrial Fe.** ES-Cu enters the cell and releases Cu in a form that is bioavailable to Ccc2. Cu from ES could be either directly transported to Ccc2 on the Golgi membrane or it could be released in the mitochondria prior to its transport to Ccc2 (indicated by *blue arrows*). Cu pumped into the Golgi lumen by Ccc2 is used for the metalation of the multicopper oxidase Fet3. This metalated Fet3 is then translocated to the plasma membrane where it oxidizes Fe^2+^ to Fe^3+^ for its import *via* Ftr1. The imported Fe is then trafficked to the mitochondria. ES, elesclomol; ES-Cu, elesclomol-copper complex.

There is a dire need for the identification, characterization, and optimization of Cu transporting drugs because currently, no effective therapy exists for Cu deficiency disorders. Our recent studies have highlighted the potential of ES, an investigational cancer drug, as a therapeutic agent for disorders of Cu deficiency (17, 18, 19). In order to translate these promising preclinical studies into repurposing ES for the treatment of Cu deficiency disorders, it is critical to determine its specificity, toxicity, and impact on the overall cellular metallome. Here, using yeast mutants, we show that in addition to the expected increase in mitochondrial Cu, ES also makes Cu bioavailable to the Golgi compartment cuproenzyme, Fet3, and thereby elevates cellular and mitochondrial Fe levels (Fig. 6).

Since ES has been used in clinical trials for the treatment of cancer, its toxicity profile has been established (38, 39, 40). However, no such information is available for ES-Cu complex. As shown in our recent study, administration of preformed ES-Cu complex is essential for treating Cu deficiency disorder, Menkes disease. Therefore, we compared the tolerability of ES and ES-Cu in parallel and found that the toxicity of ES is potentiated by the availability of Cu, with preloaded ES-Cu complex exhibiting higher toxicity than ES alone (Fig. 1*A*). Interestingly, we find that when ES treatment was given in the presence of excess Cu, the toxicity was much higher (Fig. 1*A*). Since the mechanism of action of ES is conserved from yeast to humans, this finding has an important implication for future therapeutic use of ES-Cu because in diseases like Menkes, the affected patients are given daily injections of Cu-histidinate, and thus the availability of excess Cu in the body may potentiate the toxicity of ES-Cu, if it is administered post Cu-histidinate treatment (41).

By using the more efficient Cu courier, ES-Cu, at its maximal tolerable dosage (250 nM), we sought to amplify any ES-Cu–triggered alterations in the homeostasis of cellular metals. We found that ES-Cu supplementation not only increased Cu content but also almost doubled cellular Fe content (Fig. 2). The effect of ES-Cu on Fe levels was indirect, through the activity of Ftr1-Fet3, a Cu-dependent high affinity Fe import system (Figs. 4*B* and 5*C*). This observation allowed us to dissect the mechanism by which ES distributes Cu in the intracellular compartments, specifically the Golgi lumen where Fet3 metalation takes place (42). Our study showed that Cu delivered by ES in cells is bioavailable to Ccc2, to pump Cu in the Golgi lumen. Though it was surprising that Atx1 function was dispensable for ES-mediated Cu delivery to Ccc2, there are prior studies that are consistent with this finding. For example, under the conditions of excess Cu, the ATOX1-binding domain of ATP7A is not required for Golgi Cu import (43). In two separate studies using yeast model systems, it was shown excess Cu can overcome Atx1 deficiency (44, 45). Under these conditions, it is possible that glutathione or another Cu-metallochaperone may facilitate delivery of Cu to Ccc2. In this regard, we tested the role of GRX1, which has recently been shown to transfer Cu to ATP7B *in vitro* (37). Our *in vivo* data using *grx1Δ* and *atx1Δgrx1Δ* double mutant demonstrates that Grx1 is not essential for Cu to transport to Ccc2 (Fig. 5*G*). The ability of ES to make Cu bioavailable to Ccc2 was unexpected because prior studies have shown its mitochondria-specific role in Cu delivery (21). Based on its known physical properties, it is likely that ES-Cu goes through the Golgi membrane *via* passive diffusion as it does at the plasma membrane. However, the requirement of Ccc2 for ES-mediated metalation of Fet3 suggests that ES-Cu cannot mimic the Cu transfer mechanism from Ccc2 to Fet3. Consistently, we have found that ES is not able to mimic the function of other metallochaperone such as Sco1 in delivering Cu to the mitochondrial cytochrome *c* oxidase (17).

The essential requirement of Ccc2 for ES-mediated Cu delivery to the Golgi compartment is at odds with our previously reported observations showing that ES-Cu treatment partially rescues pigmentation defects in the Menkes-affected *mo-br* mice (18). This is because pigmentation is mediated by a secretory pathway enzyme tyrosinase that receives Cu in the Golgi and melanosomes *via* the action of Ccc2 homolog ATP7A (46), which is mutated in the *mo-br* mice. Based on our results with the yeast *ccc2Δ* mutant, we would expect that a complete loss of ATP7A function would prevent ES-mediated Cu delivery to tyrosinase, which in turn would prevent melanin production. However, ES-Cu treatment did temporarily stimulate pigmentation in *mo-br* mice, which is likely due to the presence of residual activity of ATP7A in *mo-br* mice (47). Alternatively, ES-Cu could diffuse through the secretory pathway compartments, metalating tyrosinase in an ATP7A-independent manner.

Our findings described in this study have implications for possible future applications of ES-Cu in correcting defects in Fe homeostasis caused by Cu deficiency because just like in yeast, Fe and Cu metabolism are linked in humans. In fact, yeast Fet3 protein has two homologs in humans, ceruloplasmin and hephaestin, both of which play critical roles in cellular and systemic Fe metabolism (48, 49, 50). Thus, disorders of Cu metabolism are expected to have defects in Fe homeostasis due to disrupted metalation of Golgi cuproenzymes. Therefore, our studies in yeast cells warrant future investigations on the role of ES-Cu in correcting Fe homeostasis defects in mammalian systems.

## Experimental procedures

### Yeast growth

Yeast cells were cultured in either YPD (1% yeast extract, 2% peptone, and 2% dextrose) or YPGE (1% yeast extract, 2% peptone, and 3% glycerol + 1% ethanol) medium. For growth in liquid medium, yeast cells were precultured in YPD and then inoculated into either YPD or YPGE and grown to mid-log phase. ES, ES-Cu, CuCl_2_, or vehicle (DMSO) was added to growth media at the listed concentrations, and growth was monitored by measuring optical density at 600 nm (A_600_). Cultures of yeast cells with and without drug treatment were grown in YPD or YPGE for 12 or 24 h, respectively. The A_600_ value of drug-treated cells was divided by the A_600_ value of cells treated with vehicle control. This was then displayed as a percentage.

### Construction of yeast knockouts

Individual yeast *Saccharomyces cerevisiae* mutants used in this study were obtained from Open Biosystems or were constructed by one-step gene disruption using hygromycin cassette (51). All yeast strains used in this study are listed in Table 1. Authenticities of all yeast strains used in this study were confirmed by PCR-based genotyping. The primers used to disrupt *GRX1* and the primers used to confirm *grx1Δ* and *atx1Δgrx1Δ* mutants are listed in Table 2.

**Table 1:** Yeast strains used in this study

Yeast strains	Genotype	Source
BY4741 WT	*MAT*a, *his3Δ1, leu2Δ0, met15Δ0, ura3Δ0*	Dr. Miriam L. Greenberg
BY4741 *ctr1Δ*	*MAT*a, *his3Δ1, leu2Δ0, met15Δ0, ura3Δ0*, *ctr1Δ*:: *kanMX4*	Open Biosystems
BY4741 *ftr1Δ*	*MAT*a, *his3Δ1, leu2Δ0, met15Δ0, ura3Δ0*, *ftr1Δ*:: *kanMX4*	Open Biosystems
BY4741 *fet4Δ*	*MAT*a, *his3Δ1, leu2Δ0, met15Δ0, ura3Δ0*, *fet4Δ*:: *kanMX4*	Open Biosystems
BY4741 *fet3Δ*	*MAT*a, *his3Δ1, leu2Δ0, met15Δ0, ura3Δ0*, *fet3Δ*:: *kanMX4*	Open Biosystems
BY4741 *ccc2Δ*	*MAT*a, *his3Δ1, leu2Δ0, met15Δ0, ura3Δ0*, *ccc2Δ*:: *kanMX4*	Open Biosystems
BY4741 *atx1Δ*	*MAT*a, *his3Δ1, leu2Δ0, met15Δ0, ura3Δ0*, *atx1Δ*:: *kanMX4*	Open Biosystems
BY4741 *grx1Δ*	*MAT*a, *his3Δ1, leu2Δ0, met15Δ0, ura3Δ0*, *grx1Δ*::hphMX4	This study
BY4741 *atx1Δgrx1Δ*	*MAT*a, *his3Δ1, leu2Δ0, met15Δ0, ura3Δ0*, *atx1Δ*:: *kanMX4, grx1Δ*::hphMX4	This study
W303-1A WT	*MAT*a, *leu*2-3*, 112 trp1-1, can1-100, ura3-1*, *ade2-1, his3-11,15*	Dr. Miriam L. Greenberg
SY025	*MATa hoΔ::[tetO*_*2*_*pr-GFP, URA3] can1Δ::*GMToolkit-a *lyp1Δ, his3Δ1, leu2Δ0, met15Δ0, ura3Δ0*	Dr. Frederick P. Roth
ABC_16_ Monster	*adp1Δ, snq2Δ, ycf1Δ, pdr15Δ, yor1Δ, vmr1Δ, pdr11Δ, nft1Δ, bpt1Δ, ybt1Δ, ynr070wΔ, yol075cΔ, aus1Δ, pdr5Δ, pdr10Δ, pdr12Δ, can1Δ::*GMToolkit-a *lyp1Δ, his3Δ1, leu2Δ0, met15Δ0, ura3Δ0*	Dr. Frederick P. Roth

**Table 2:** Oligonucleotides used in this study

Oligonucleotide used	Sequence (5′–3′)
Confirmation of *grx1Δ* mutant	AGTGAGCTGTCTACAGATAACGAGC
	TCTTAAAGTAATGGGCCAAGTAAAA
Generation of *grx1Δ* mutant	ATAATTATACAAATAGACAAAACCTCAGAAGGAAAAAAAATGCGTACGCTGCAGGTCGAC
	TTATAAACCTGTGTGCATGGAAAAAACTTTGTCTGCCCTTAATCGATGAATTCGAGCTCG

### Mitochondrial isolation

Mitochondria were isolated as described previously (52). Briefly, 0.5 to 2.5 g of cell pellet was incubated in DTT buffer (0.1 M Tris–HCl, pH 9.4, 10 mM DTT) at 30 °C for 20 min. The cells were then pelleted by centrifugation at 3000*g* for 5 min, resuspended in spheroplasting buffer (1.2 M sorbitol, 20 mM potassium phosphate, pH 7.4) at 7 ml/g, and treated with 3 mg zymolyase (US Biological Life Sciences) per gram of the cell pellet for 45 min at 30 °C. Spheroplasts were pelleted by centrifugation at 3000*g* for 5 min and then homogenized in homogenization buffer (0.6 M sorbitol, 10 mM Tris–HCl, pH 7.4, 1 mM EDTA, 1 mM PMSF, and 0.2% [w/v] BSA [essentially fatty acid-free, Sigma-Aldrich]) with 15 strokes using a glass Teflon homogenizer with pestle B. After two centrifugation steps of 5 min at 1500*g* and 4000*g*, the final supernatant was centrifuged at 12,000*g* for 15 min to pellet mitochondria. Mitochondria were resuspended in Sucrose–EDTA-Mops mitochondria isolation buffer (250 mM sucrose, 1 mM EDTA, 10 mM Mops–KOH, pH 7.2, containing 1× protease inhibitor cocktail from Roche).

### Inductively coupled plasma-mass spectrometry

Cellular and mitochondrial Cu and Fe levels were measured by ICP-MS using the NexION 300D instrument from PerkinElmer, Inc. Briefly, 80 to 300 mg of intact yeast cells were washed twice with 1 ml of ultrapure metal-free water containing 100 μM EDTA (TraceSELECT; Sigma-Aldrich), followed by two more washes with ultrapure water to eliminate EDTA. For mitochondrial samples, the same procedure was performed using 300 mM mannitol (TraceSELECT; Sigma-Aldrich) to maintain mitochondrial integrity. After washing, the samples were weighed, digested with 40% (w/v) nitric acid (TraceSELECT; Sigma-Aldrich) at 90 °C for 18 h, followed by 6 h digestion with 0.75% H_2_O_2_ (Sigma-Supelco), and then diluted in ultrapure water. For Zn, Mg, and Ca, digested samples were further diluted by 10- to 100-fold to ensure that the concentration of these ions were within the linear range of the calibration curve. Calibration curves were generated by serially diluting a premixed standard solution (BDH Alistar plus or Milipore sigma ICP multielement standard solution VIII).

### Fet3 oxidase assay

Fet3p oxidase activity was measured by an in-gel assay using p-phenylenediamine dihydrochloride as a substrate (48, 53, 54). Cells were cultured to mid-log phase in YPD with 160 μM of Fe chelator bathophenanthroline disulfonate (Sigma-Aldrich) because bathophenanthroline disulfonate enhances the expression of Fet3p to easily detectable levels. Cells were incubated in the Tris–HCl buffer (50 mM, pH 7.4) containing protease inhibitor cocktail (Roche), PMSF (1 mM) (Sigma-Aldrich) before disrupting them by vortexing (1 min vortexing followed by 1 min incubation on ice, for a total of 8 times) in the presence of glass beads. After removing unbroken cells and glass beads by centrifugation at 300*g* for 3 min, membrane fraction was obtained by centrifugation at 21,000*g* for 30 min. Membrane pellets were washed with fresh buffer, incubated with the same buffer containing Triton X-100 (1% final concentration) on ice for 30 min with vortexing every 5 min, and then cleared by centrifugation (21,000*g*, 30 min). Protein concentration was measured by the bicinchoninic acid assay (Thermo Fisher Scientific).

Samples were resuspended in Laemmli sample buffer without DTT and subjected to 4% to 20% SDS-PAGE. The gels were soaked in 50 ml of a solution containing 10% glycerol and 0.05% Triton X-100 for 20 min. Gels were then cut at the 38 kDa mark. The bottom portion of the gel was imaged using a stain-free filter (BIO-RAD ChemiDoc MP). The top portion of the gel was placed in 50 ml of 100 mM sodium acetate (pH 5.7) solution containing 0.1% p-phenylenediamine dihydrochloride (Sigma-Aldrich) for 30 min in dark before imaging. Quantification of Fet3 activity was determined by densitometric analysis performed using ImageJ software.

### Statistical analysis

Statistical analysis on bar charts was conducted using two-sided Student’s *t* test. Experiments were performed in three biological replicates, where biological replicates are defined as experiments performed on different days with different clones. Error bars represent SD.

## Data availability

All data is available in the main text or the supporting information.

## Supporting information

This article contains supporting information.

## Conflict of interest

The authors’s university (Texas A&M) has entered into a licensing agreement with Engrail Therapeutics for the development of elesclomol:copper as a therapeutic agent for the disorders of copper metabolism. Engrail therapeutics also sponsors certain aspects of elesclomol research in the author’s laboratory. The authors declare that they have no conflicts of interest with the contents of this article.

## Supporting information

Suplemental infomation
