# Supplementary material for: Elesclomol elevates cellular and mitochondrial iron levels by delivering copper to the iron import machinery
Source: J Biol Chem. 2022 Jun 14;298(7):102139. doi: 10.1016/j.jbc.2022.102139 (PMC9270252; doi:10.1016/j.jbc.2022.102139)
Supplement: Suplemental infomation [file mmc1.pdf]

Supporting Information

Title: Elesclomol elevates cellular and mitochondrial iron levels by delivering copper to the iron import machinery

Natalie M. Garza, Mohammad Zulkifli, Vishal M. Gohil*

Table of Contents

Supplementary Figure 1……………………………………………………………S-2
Supplementary Figure 2……………………………………………………………S-3
Supplementary Figure 3……………………………………………………………S-4

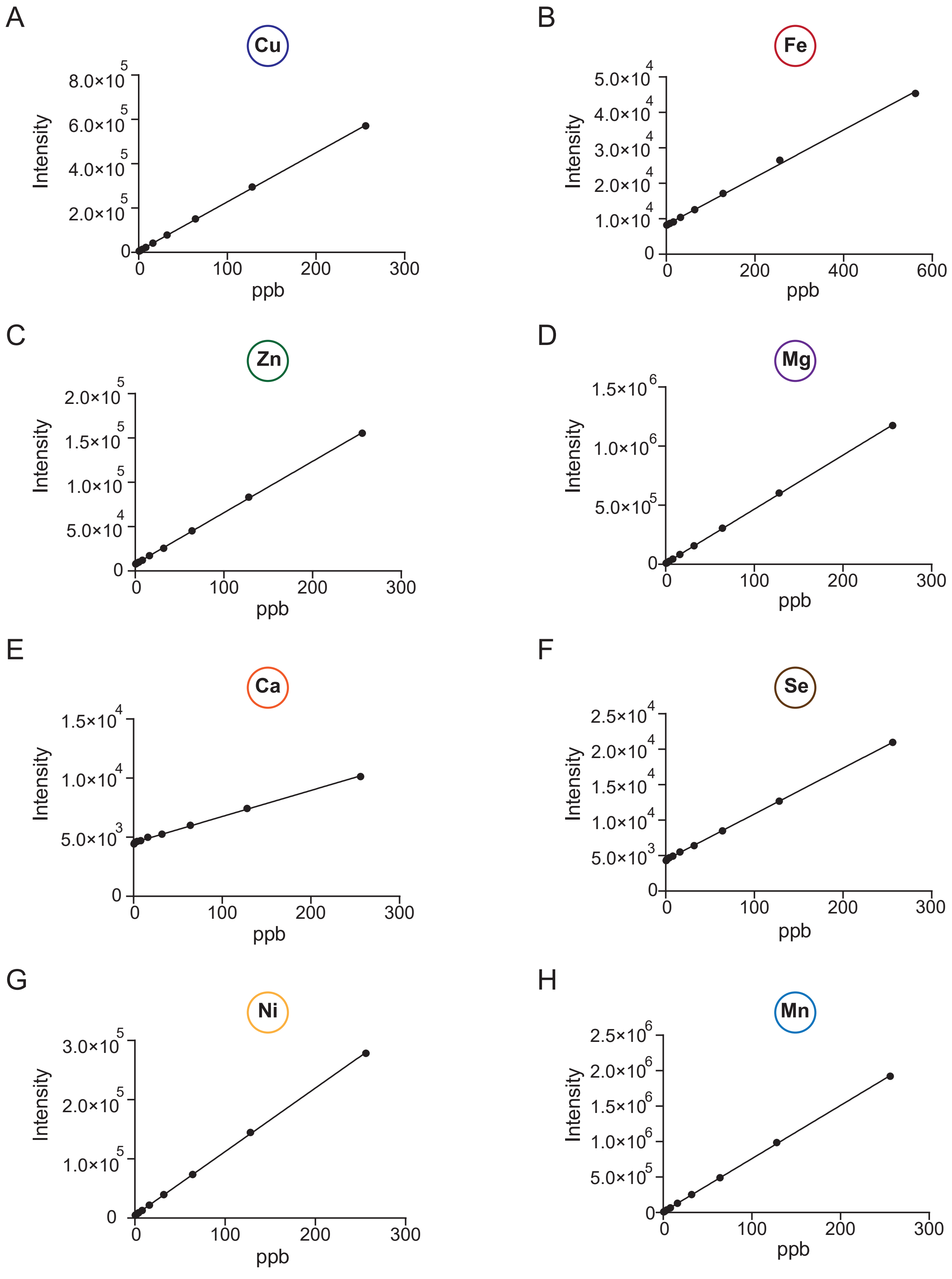

Supplementary Figure S1. ICP-MS calibration curves of different cellular metals. Calibration curves were generated by measuring the raw intensity of the signal generated by each of the indicated ions at given concentrations. These calibration curves were utilized to calculate the concentrations of cellular metal content reported in figure 2.

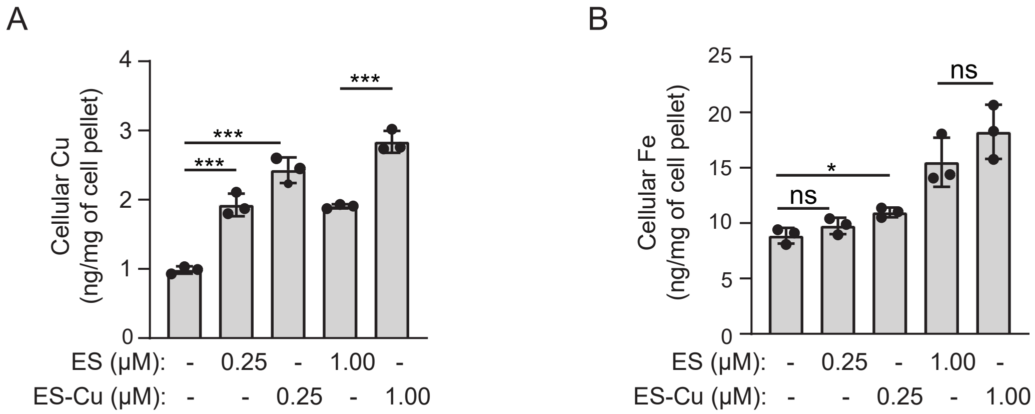

Supplementary Figure S2. ES-Cu stimulates increase in cellular Fe levels in yeast strain W303-1A. Cells were grown in YPD with indicated concentrations of ES or ES-Cu for 20 hours before measuring cellular A) Cu and B) Fe levels by ICP-MS. The data represent the average ± SD of three biological replicates. ***p < 0.001, *p < 0.05, ns= not significant.

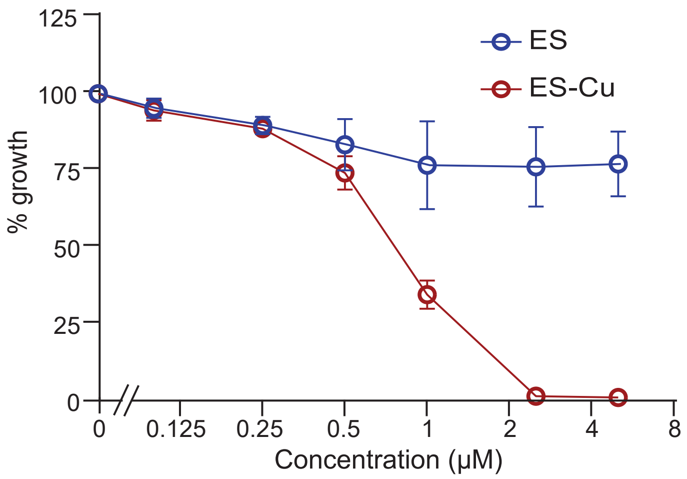

Supplementary Figure S3. Yeast cells are more sensitive to ES-Cu as compared to ES in respiratory growth media. BY4741 WT yeast cells were cultured in YPGE medium at 30°C in the presence of increasing concentrations (0.1 to 5 μM) of either ES or ES-Cu. The cell density was measured spectrophotometrically after 24 hours of growth at 600 nm. Percent of growth was calculated by comparing the optical density of each culture to that of WT with vehicle alone. The data represent the average of three biological replicates ± SD.
